# Integrated traditional Chinese and Western medicine in the prevention and treatment of non-alcoholic fatty liver disease: future directions and strategies

**DOI:** 10.1186/s13020-024-00894-1

**Published:** 2024-02-03

**Authors:** Xin Ding, Xu He, Bulang Tang, Tian Lan

**Affiliations:** 1 School of Pharmacy, Guangdong Pharmaceutical University, Guangzhou Higher Education Mega Center, 280 Wai Huan Dong Road, Guangzhou, 510006 China; 2 School of Pharmacy, Harbin Medical University, Harbin, 150086 China

**Keywords:** Non-alcoholic fatty liver disease, Western medicine, Traditional Chinese medicine, Pathogenesis, Treatment strategies

## Abstract

Traditional Chinese medicine (TCM) has been widely used for several centuries for metabolic diseases, including non-alcoholic fatty liver disease (NAFLD). At present, NAFLD has become the most prevalent form of chronic liver disease worldwide and can progress to non-alcoholic steatohepatitis (NASH), cirrhosis, and even hepatocellular carcinoma. However, there is still a lack of effective treatment strategies in Western medicine. The development of NAFLD is driven by multiple mechanisms, including genetic factors, insulin resistance, lipotoxicity, mitochondrial dysfunction, endoplasmic reticulum stress, inflammation, gut microbiota dysbiosis, and adipose tissue dysfunction. Currently, certain drugs, including insulin sensitizers, statins, vitamin E, ursodeoxycholic acid and betaine, are proven to be beneficial for the clinical treatment of NAFLD. Due to its complex pathogenesis, personalized medicine that integrates various mechanisms may provide better benefits to patients with NAFLD. The holistic view and syndrome differentiation of TCM have advantages in treating NAFLD, which are similar to the principles of personalized medicine. In TCM, NAFLD is primarily classified into five types based on clinical experience. It is located in the liver and is closely related to spleen and kidney functions. However, due to the multi-component characteristics of traditional Chinese medicine, its application in the treatment of NAFLD has been considerably limited. In this review, we summarize the advances in the pathogenesis and treatment of NAFLD, drawn from both the Western medicine and TCM perspectives. We highlight that Chinese and Western medicine have complementary advantages and should receive increased attention in the prevention and treatment of NAFLD.

## Introduction

NAFLD is the most common chronic liver disease worldwide with a global prevalence of 32.4% [1]. This disease encompasses a spectrum of liver disorders ranging from non-alcoholic fatty liver (NAFL), characterized by simple steatosis, to non-alcoholic steatohepatitis (NASH), characterized by hepatocyte steatosis accompanied by inflammation, hepatocyte ballooning, progressive fibrosis, and even further progression to cirrhosis and hepatocellular carcinoma (HCC) [2]. The multiple-hits hypothesis suggests that insulin resistance, lipotoxicity, endoplasmic reticulum stress, inflammation, mitochondrial dysfunction, oxidative stress, gut microbiota, and genetics synergistically contribute to the development of NAFLD [3]. Furthermore, NAFLD frequently coexists with many metabolic diseases such as type 2 diabetes (T2D), which is associated with an increased risk of adverse outcomes in NAFLD patients [4]. Although many clinical and experimental studies have been performed, the detailed molecular mechanisms involved in the pathogenesis of NAFLD remain to be elucidated. Lifestyle modifications, including weight loss, a Mediterranean diet, and physical activity, are recognized as the most effective non-drug treatment strategies, while there are still no specific drugs available for NAFLD in clinical settings due to its complex pathogenesis [2]. However, increasing evidence indicates that incorporating drugs with different mechanisms and personalized medicine may improve treatment efficacy in NAFLD patients [5].

**Fig. 1:**
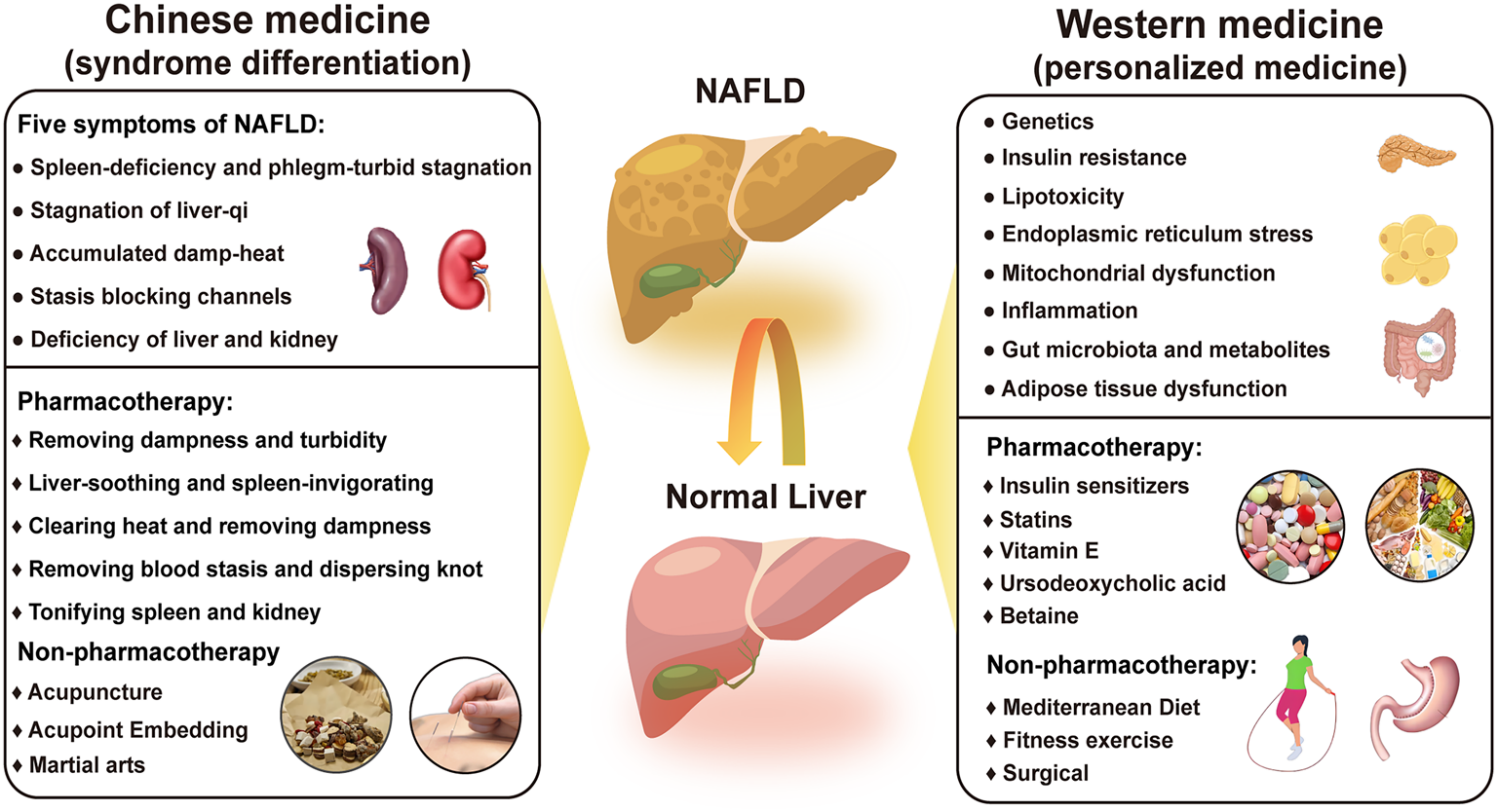
The pathogenesis and treatment strategies for NAFLD in Western medicine and TCM

Traditional Chinese medicine (TCM) has been widely used in China and is increasingly recognized as a complementary and alternative form of modern medicine, including herbal medicine, acupuncture, and other physical therapies such as massage [6]. Holistic view and syndrome differentiation are the theoretical guidance of TCM and reflect system physiology approaches and personalized medicine in Western medicine [7], demonstrating their advantages in NAFLD treatment [8]. In TCM, NAFLD pathogenesis is primarily attributed to deficiency of the spleen (Pi) or kidney (Shen), stagnation of liver qi, and internal retention of phlegm, dampness, and blood stasis [9]. Consistently, the theoretical guidance of NAFLD treatment includes removing phlegm and blood stasis, nourishing the liver and kidneys, relaxing the liver and spleen, eliminating moisture, and clearing heat [10]. However, the diagnosis and treatment of NAFLD in TCM are subject to subjectivity and complexity. Therefore, it is necessary to establish standardization and objective quantitative analysis methods in combination with Western medicine to improve the clinical efficacy of NAFLD treatment [11]. This review presents the current progress in the pathogenesis and treatment of NAFLD in Chinese and Western medicine (Fig. 1).

## The pathogenesis of NAFLD in Western medicine

**Fig. 2:**
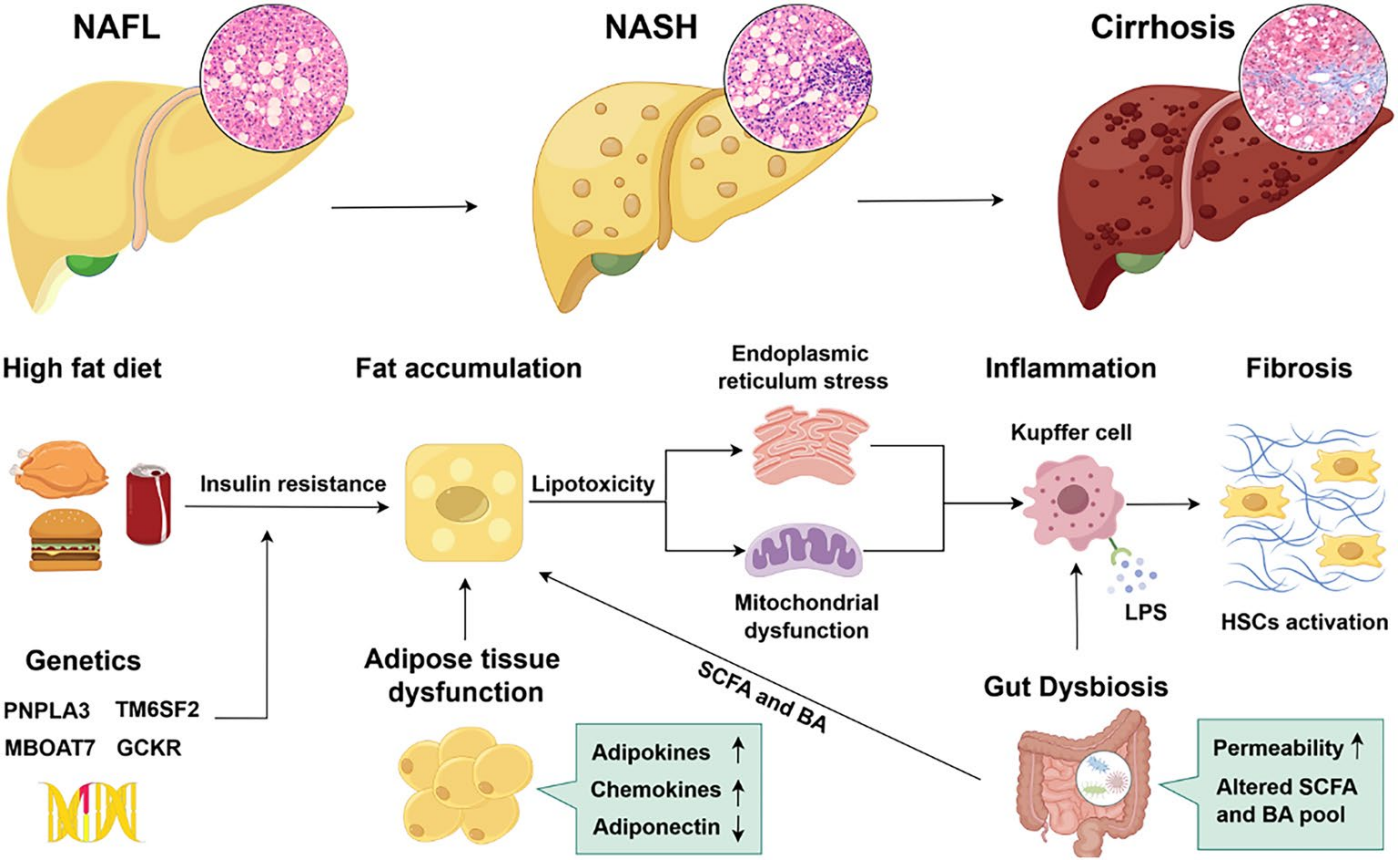
The pathogenesis of NAFLD in Western medicine. The risk of developing NAFLD is determined by environmental and genetic factors. High-fat diet-induced insulin resistance and disruptions in hepatic lipid metabolism, such as imbalances in lipid synthesis and catabolism or impaired lipid transport, result in the excessive accumulation of lipids in hepatocytes. Concurrently, factors such as lipotoxicity, endoplasmic reticulum stress and mitochondrial dysfunction contribute to the inflammation and apoptosis of overloaded hepatocytes. Inflammation can exacerbate hepatocyte injury and stimulate hepatic stellate cell activation, leading to hepatic fibrosis. Additionally, the interplay between the liver, adipose tissue and the gut contributes to metabolic dysregulation and inflammation in NAFLD

The two-hit theory was proposed in 1998, which posits that the first hit, steatosis, increases liver sensitivity to the second hits that mediate liver injury. However, with the emergence of new findings, the traditional two-hit theory is no longer sufficient to explain the complex pathogenesis of NAFLD, and multiple parallel strikes are gradually replacing the second strike theory to explain the pathogenesis of NAFLD. Such hits include genetic factors, insulin resistance, lipotoxicity, mitochondrial dysfunction, endoplasmic reticulum stress, inflammation, gut microbiota dysbiosis, and adipose tissue dysfunction [12, 13] (Fig. 2). However, the interactions and synergies between these mechanisms and their changing patterns in NAFLD development require further clarification.

### Genetics

Genetic and epigenetic changes interact with environmental factors to determine susceptibility to NAFLD [14]. A cross-sectional analysis indicated that the risk of severe liver fibrosis was 12-fold higher in first-degree relatives of patients with NAFLD-related cirrhosis than in the general population [15]. Moreover, the incidence of NAFLD in the United States has significant racial and ethnic disparities [16]. Genome-wide association studies in recent years have revealed the important role of genetic polymorphisms in the progression of NAFLD.

The I148M variant disrupts the phospholipase activity of patatin-like phospholipase domain-containing 3 (PNPLA3), a triglyceride lipase, thereby interfering with lipid catabolism and promoting hepatic fat accumulation to increase the risk of liver-related mortality in patients with NAFLD [17, 18]. The E167K variant of transmembrane 6 superfamily member 2 (TM6SF2) decreases the secretion of very low-density lipoprotein (VLDL) to favor hepatic fat accumulation in NAFLD patients but confers cardiovascular protection by reducing the amount of circulating lipids [19, 20]. The rs641738 variant downregulates the expression of membrane-bound O-acyltransferase domain-containing 7 (MBOAT7) and reduces the levels of phosphatidylinositol species with arachidonoyl side chains in hepatocytes, which might contribute to fibrosis independent of inflammation in NAFLD [21, 22]. The P446L variant of glucokinase regulatory protein (GCKR) increases hepatic glucose uptake and the production of malonyl-CoA, thereby promoting lipid accumulation in the liver by increasing lipogenesis substrates and blocking fatty acid oxidation [23, 24]. In addition, several other genetic determinants of NAFLD have been identified, including hydroxysteroid 17-beta dehydrogenase 13 (HSD17B13) [25], phosphatase 1 regulatory subunit 3B (PPP1R3B) [26], immunity-related GTPase M (IRGM) [27] and Lpin1 [28].

### Insulin resistance

Insulin, a hormone, regulates cellular functions, stimulates sugar transport, and thus governs cell growth, energy balance and gene expression, and inhibits lipolysis in adipose tissue. It binds to its receptors, initiating the phosphorylation of various downstream substrates, such as insulin receptor substrate (IRS)-1, -2, -3, and -4, thereby activating the insulin signaling pathway [29]. IRS-1 and IRS-2 phosphorylate phosphoinositide 3-kinase (PI3K) and activate protein kinase B (AKT), resulting in the translocation of glucose transporter protein 4 (GLUT4). This process enhances glucose uptake and modulates forkhead transcription factors, increasing lipid gene expression and decreasing glucose xenobiotic gene expression [30]. Molecular substances associated with NAFLD interfere with insulin signaling connections and exacerbate insulin resistance, including non-esterified fatty acids, tumor necrosis factor, nuclear factor-κB (NF-κB), suppressor of cytokine signaling (SOCS), Jun amino-terminal kinase 1 (JNK1), and cytochrome P4502E1 [31]. Furthermore, substantial evidence suggests that IR may precede NAFLD. Reduced metabolic activity may protect against excessive transport of free fatty acids (FFAs) to the liver [32].

### Lipotoxicity

The core mechanisms of NASH are endoplasmic reticulum stress, oxidative stress, and inflammatory responses caused by free cholesterol, FFAs, and their metabolites. The conventional view is that triglycerides in hepatocytes promote lipid peroxidation, oxidative stress, inflammation, and fibrosis and are drivers of NAFLD progression. However, this viewpoint is increasingly challenged because triglycerides may antagonize lipotoxicity. A study revealed that inhibiting triglyceride synthesis in the mouse liver reduced hepatic steatosis but exacerbated liver damage and fibrosis [33]. Triglyceride (TG) deposition in the mouse liver was insufficient to induce insulin resistance and liver inflammation [34]. These results suggest that triglycerides may not be the primary type of lipid that promotes NAFLD disease progression.

Studies have shown that palmitic acid (C16:0) and stearic acid (C18:0), which are abundant in animal fat and dairy products, can promote the progression of NAFLD. Furthermore, ceramides can increase mitochondrial membrane permeability to promote cell death by activating JNK activity [35]. The accumulation of lysophosphatidylcholine in hepatocytes can cause apoptosis by activating the death receptor DR5 while promoting macrophage activation through pro-inflammatory exosomes [36]. The increase in excess free cholesterol in cells can disrupt the fluidity of mitochondrial membranes, leading to mitochondrial malfunction and oxidative stress. The accumulation of cholesterol crystals in hepatocytes can activate inflammasome and promote the production of inflammatory factors such as IL-1β and TNF-α [37]. Therefore, in some animal models of NASH, high cholesterol and free fatty acid levels promote NASH phenotype development [38].

### Endoplasmic reticulum stress

The endoplasmic reticulum (ER) is the major site of protein and lipid synthesis in hepatocytes [39]. The accumulation of misfolded or unfolded proteins leads to ER stress and the activation of the unfolded protein response (UPR), which mainly relies on three transmembrane ER stress sensors, inositol-requiring enzyme 1 (IRE1), PKR-like ER kinase (PERK) and activating transcription factor (ATF6), to restore homeostasis [40].

IRE1α in NAFLD demonstrates a dichotomous impact. IRE1α facilitates serine palmitoyltransferase gene transcription via XBP1s, leading to the release of extracellular vesicles (EVs) from hepatocytes, thereby recruiting macrophages to foster hepatic inflammation and injury in NASH mice [41]. Conversely, S-nitrosylation of IRE1α impedes its degradation of miR-34 and miR-200, which are pivotal microRNAs that govern hepatic lipid metabolism [42]. This finding was further corroborated by liver-specific IRE1α knockout, which exacerbates liver fibrosis and inflammation in high-fat diet-induced mice [43].

Activation of the PERK-eIF2α pathway leads to the up-regulation of UPR target genes, including the proapoptotic protein C/EBP homologous protein (CHOP). Interestingly, CHOP-deficient hepatocytes exhibit attenuated ER stress-induced cell death [44], while CHOP-dependent macrophage apoptosis protects against NAFLD [45]. Moreover, salubrinal, an eIF2α inhibitor, has been shown to have hepatoprotective effects on high-fat diet-induced obese mice [46].

Reduced endoplasmic reticulum (ER) membrane fluidity, which selectively activates ATF6α, is implicated in NASH [47]. ATF6p50, a cleaved form of ATF6α, may indirectly suppress lipogenic genes by inducing CHOP through dominant-negative inhibition of C/EBPα, leading to diminished fatty acid oxidation and lipoprotein secretion [48].

In addition, deletion of sterol regulatory element-binding protein (SREBP) cleavage-activating protein (SCAP) exacerbates ER stress and liver injury through impairing FA synthesis and discerning FA incorporation into phosphatidylcholine [49]. SID1 transmembrane family member 2 (SIDT2) deficiency induces ER stress and results in hepatic steatosis by upregulating SREBP1, sterol regulatory element binding transcription factor 1 (SREBF1) and fatty acid biosynthesis [50]. Forkhead box A3 (FOXA3) is selectively induced by XBP1s during endoplasmic reticulum stress and directly orchestrates Period1 (Per1) expression, thereby enhancing the expression of the lipogenic gene Srebp1c [51].

### Mitochondrial dysfunction

Mitochondria, which are abundant in hepatocytes and reliant on oxygen for adenosine triphosphate (ATP) synthesis, play a pivotal role in the metabolic functions of the liver. During fatty acid catabolism, FFAs are initially converted to fatty acyl-CoA in the cytosol of hepatocytes and subsequently transported into mitochondria by carnitine palmitoyl-transferase 1 (CPT1). β-Oxidation efficiently breaks down FFAs into acetyl-CoA, which is subsequently metabolized to CO_2_ and H_2_O via the tricarboxylic acid (TCA) cycle [52]. However, an imbalance between saturated and unsaturated FAs leads to reduced levels of linoleic and arachidonic acids in mitochondrial phospholipids, resulting in decreased CPT1 activity [53]. The electron transport chain (ETC) is situated in the inner mitochondrial membrane and consists of five complexes, including complexes I–IV. High-fat diets increase calcium uptake to modulate the influx of sodium into the mitochondrial matrix via sodium/calcium exchangers, resulting in a reduction in inner mitochondrial membrane fluidity and negatively impacting mitochondrial ATP production [54]. Consistently, studies have shown increased mitochondrial mass and reduced efficiency of respiratory chain complexes, accompanied by elevated mitochondrial uncoupling and leakage activity in patients with NAFLD [55]. Furthermore, a recent study reported the presence of two distinct mitochondrial populations in the liver, which are named cytoplasmic mitochondria and lipid droplet-associated mitochondria. Lipid droplet-associated mitochondria exhibit increased fatty acid oxidation, as indicated by elevated levels of phosphorylated ACC2 (pACC2), Mitofusin 2 protein (MFN2) and CPT1 activity, but are damaged by fatty acid oxidation in an NAFLD rat model [56]. Furthermore, when mitochondrial β-oxidation is impaired, alternative peroxisomal and cytochrome oxidation pathways for FFAs are activated, resulting in the generation of substantial levels of reactive oxygen species (ROS) and oxidative stress [57].

### Inflammation

A crucial pathological feature in the progression of NAFLD is chronic, persistent, low-grade non-infectious inflammation. Elevated levels of FFAs and their lipotoxicity, IR, ER stress and adipose tissue dysfunction activate and sustain the production and release of hepatic pro-inflammatory cytokines [58]. High caloric intake causes the expansion of adipose depots, which is associated with increased infiltration of macrophages [59]. IR is closely related to the inflammatory response and is now considered a chronic subclinical inflammatory process [60]. Under physiological conditions, the IRS undergoes tyrosine phosphorylation. Moreover, inflammatory factors activate a series of kinases to induce serine/threonine phosphorylation in the IRS, which prevents normal tyrosine phosphorylation and leads to the loosening of the binding of the IRS to the insulin receptor and the inability of the IRS to activate its downstream substrate phosphatidylinositol 3-kinase (PI3-K), thus interfering with the passage of IR/IRS/PI insulin signaling [60]. ER stress plays an essential role in the activation of the NLRP3 inflammasome, which results in the development of NAFLD. The expression of various inflammatory cytokines, such as TNF-α, IL-1β and MCP-1, is induced by the NLRP3 inflammasome through the JNK/AP-1 and IKK/NF-κB pathways [61]. Furthermore, overnutrition increases intestinal permeability and results in bacterial and lipopolysaccharide (LPS) migration via the portal vein to the liver, where they react with Toll-like receptors (TLRs) and activate NF-κB to induce the secretion of inflammatory cytokines, thereby maintaining and aggravating hepatic inflammation [62]. Inflammatory cytokines activate Kupffer cells and hepatic stellate cells (HSCs), which exacerbate the inflammatory response and lead to hepatic damage and fibrosis [58].

### The gut microbiota and metabolites

Both the liver and intestine originate from the ventral foregut endoderm. They are intrinsically linked in anatomy and biological function [63]. The liver and the intestine are connected through the portal vein, through which 70–75% of the blood supply to the liver passes, while the portal vein originates from the intestinal capillaries. Moreover, a range of bacteria, bacterial metabolites, and environmental toxins produced in the intestine can reach the liver through the portal vein [64], making the liver the most accessible place for intestinal bacteria and their derivatives. The tight junctions of intestinal epithelial cells act as a natural barrier to bacteria and their metabolites [65]. Antigens from pathogenic microorganisms or foods are recognized by dendritic cells in the intestinal epithelium to activate adaptive T cells. Pathogen-associated molecular patterns (PAMPs), such as lipopolysaccharide (LPS), peptidoglycan, and flagellin, are recognized by Toll-like receptors (TLRs) and Nod-like receptors (NLRs). PAMPs activate HSCs involved in the fibrotic process, but Kupffer cells respond more strongly to LPS than HSCs [66].

Primary bile acids (BAs) are synthesized by the liver and secreted into the gallbladder. These compounds are released into the duodenum after feeding and are metabolized by intestinal bacteria to secondary bile acids and then reabsorbed into the portal vein, where most of the molecules are captured by the liver for recirculation [67]. The most crucial bile acid receptors in NAFLD are the farnesyl X nuclear receptor (FXR), which is activated mainly by primary bile acids, and transmembrane G-protein-coupled receptor 5 (TGR5), which is activated primarily by secondary bile acids [68]. FXRs are members of the nuclear receptor superfamily and play a role in hepatic bile acid uptake and transport. Hepatic TG and cholesterol levels are significantly increased in FXR-deficient mice [69]. However, intestinal bacteria interact with FXRs to reduce hepatic lipogenesis by reducing glucagon-like peptide 1 (GLP-1) expression in the gut and increasing hepatic gluconeogenesis [70]. TGR5 activation induces the transcription factor cAMP-responsive element binding protein (CREB) and enhances the production of GLP-1 to regulate glucose and energy metabolism [71].

Short-chain fatty acids (SCFAs) are volatile fatty acids produced mainly by the fermentation of soluble dietary fiber and indigestible carbohydrates by intestinal microorganisms [72]. SCFAs play various roles in body metabolism, such as regulating appetite and energy expenditure, stimulating insulin sensitivity, and activating adenosine monophosphate in the liver and skeletal muscle [73]. One of the mechanisms by which SCFAs affect hepatic adiposity and adipose tissue accumulation is the regulation of insulin sensitivity in adipose tissue through G-protein coupled receptor 43 (GPR43). In terms of insulin sensitivity, GRP43 in adipose tissue is activated by SCFAs to promote energy expenditure and inhibit adipose tissue accumulation [74]. Moreover, the recognition of SCFAs by the intestine can cause the release of insulin-sensitive peptide tyrosine (PYY) and GLP-1, which inhibits gastrointestinal motility and prolongs gastrointestinal transit time [75].

### Adipose tissue dysfunction

Adipose tissue is an important site of energy metabolism and an essential endocrine organ of the body. Increasingly, studies have shown that fatty endocrine factors can regulate the function of other metabolic organs, thereby regulating metabolic homeostasis throughout the body. In obesity, insulin resistance, and metabolic syndrome, fatty tissue becomes chronically inflamed and secretes increased levels of inflammatory factors such as IL-1β, TNF-α, and IL-6, which stimulate liver inflammation and disrupt glycolipid metabolism through the body circulation [58]. Moreover, adiponectin from adipose tissue inhibits insulin resistance, reduces hepatic steatosis, and has anti-inflammatory, anti-apoptotic, and insulin-sensitizing effects in NAFLD [76].

Leptin may play a dual role in the pathogenesis of NAFLD. In animal models, leptin has been found to alleviate hepatic steatosis by inhibiting the de novo synthesis of hepatic lipids (DNL) in the early stages of NAFLD disease, but promoting inflammation and hepatic fibrosis in the progressive stages of NAFLD disease [77, 78]. A recent study demonstrated that leptin in the brain increases very-low-density lipoprotein triglyceride (VLDL-TG) levels and reduces hepatic lipid content through the vagus nerve [79]. Lipocalin-2 activates HSCs to secrete matrix metalloproteinase 9 (MMP9) in leptin-deficient obesity, thereby promoting the transition from simple steatosis to NASH [80]. Consistently, a study reported that a decrease in lipocalin levels may be associated with the development of NAFLD [81]. Neuregulin 4 (Nrg4), from brown adipose tissue, negatively regulates de novo lipogenesis mediated by liver X receptor (LXR) and SREBP1c [82]. In addition, Nrg4 restrains tumor-associated macrophage (TAM)-like macrophages and the exhaustion of cytotoxic CD8^+^ T cells to suppress NASH progression to liver cancer [83].

## The pathogenesis of NAFLD in TCM

NAFLD is a term used in Western medicine, but there is no direct equivalent of this disease in TCM. However, TCM has recorded this disease for a long time. *Su Wen* registered, “Excessive diet will damage the gastrointestinal tract”. According to its symptoms and pathogenesis, NAFLD can be recognized as a hepatic syndrome such as distention and fullness, phlegm syndrome, turbidity, hypochondriac pain, a lump at the left hypochondrium and damp obstruction disease [8]. In TCM, the primary causes of NAFLD are identified as emotional disorders, irregular diet, work stress, chronic diseases and congenital deficiencies. It is located mainly in the liver and is closely related to spleen and kidney functions [8].

There are many theories on the treatment of NAFLD in TCM, primarily based on the theory of liver and spleen [9]. Spleen deficiency can result in impaired organ transformation and transportation functions, resulting in gastrointestinal disturbances. Correspondingly, disturbance in the gut microbiota can lead to functional impairments in the digestive system, resulting in spleen deficiency syndrome [84]. Physiologically, the transportation function of the spleen depends on the maintenance of liver qi, while the drainage and blood storage functions of the liver require vital qi produced by spleen metabolism. This interplay reflects the roles and relationships between the liver and spleen in transportation, blood production and blood storage.

Most related research emphasizes NAFLD treatment based on the liver and spleen theories, while the importance of the kidney is frequently overlooked. In *Su Wen Yin Yang Xiang Da Lun*, the theory of reciprocal generation of the essence and blood between the liver and kidney is discussed: “North generates cold, cold generates water, water generates salt, salt generates kidney, kidney generates bone marrow, marrow generates liver.” This elucidates the liver-kidney relationship, forming the basis of the “liver and kidney share the same origin” theory [85]. One of the fundamental theories of TCM is that the kidney collects essence, and the liver stores blood, then they continuously transform within the body along with qi. Sufficient blood can nourish and continuously enrich kidney essence, which is the physiological relationship between the liver and kidney.

### Identification of NAFLD patterns in TCM

NAFLD can be classified into five main types based on clinical experience: spleen-deficiency and phlegm-turbid stagnation; stagnation of liver-qi; accumulated damp-heat; stasis blocking channels and deficiency of liver and kidney [86, 87].

### Spleen-deficiency and phlegm-turbid stagnation

Primary symptoms include fullness in the right hypochondrium. Secondary symptoms include obesity, bodily heaviness, lethargy, chest tightness, dizziness and nausea.

### Stagnation of liver-qi

Primary symptoms consist of congestion or wandering pain in the right hypochondrium, often accompanied by depression due to irritability. Secondary symptoms include abdominal distension, diarrhea, abdominal pain, fatigue, and chest tightness.

### Accumulated damp-heat

The primary symptoms involve fullness in the right hypochondrium. Secondary symptoms include nausea, vomiting, jaundice, chest fullness, and body heaviness.

### Stasis blocking channels

Primary symptoms include a lump or tingling pain in the right abdomen. Secondary symptoms include anorexia, chest tightness, and a dark complexion.

### Deficiency of liver and kidney

Primary symptoms present as pain in the right hypochondrium. Secondary symptoms include physical weakness, lumbar and knee soreness, frequent nocturnal urination, and diarrhea.

## Pharmacological Treatment of NAFLD in Western Medicine

In Western medicine, although there are currently no approved drugs for NAFLD, some drugs, such as insulin sensitizers, statins, vitamin E, ursodeoxycholic acid and betaine, are beneficial for the clinical treatment of NAFLD. Among these, insulin sensitizers, statins and vitamin E are primarily recommended in clinical practice guidelines for NAFLD [88, 89]. Here, we summarize the research progress related to these drugs in NAFLD treatment.

### Insulin sensitizers

Insulin sensitizers are applicable in patients with both NAFLD/NASH and type 2 diabetes mellitus. Metformin is the first-line treatment for type 2 diabetes, which elevates hepatic glucose output while reducing insulin-stimulated glucose uptake in peripheral tissues and fatty acid oxidation in adipose tissue [90]. A study demonstrated that metformin can reverse transaminase abnormalities, steatosis, and inflammation in NAFLD and NASH patients [91]. Metformin alleviates hepatic steatosis by restoring SIRT1-mediated autophagy and inducing tristetraprolin to regulate lipophagy and necroptosis in hepatocytes [92, 93]. A recent study demonstrated that metformin can rescue the impairment of hepatic CD8^+^ T cell metabolism and the efficacy of anti-PD-1 therapy against NASH-related liver cancer [94]. Furthermore, combination of metformin, both liraglutide and sitagliptin can reduce body weight, intrahepatic lipids, and visceral adipose tissue and improve glycemic control in patients with T2DM and NAFLD under conditions of inadequate glycemic control caused by metformin [95]. However, long-term metformin treatment might promote NAFLD progression in leptin-insensitive individuals [96].

Thiazolidinediones (TZDs) are a class of oral antidiabetic drugs. They induce the nuclear transcription factor peroxisome proliferator-activated receptor-γ (PPAR-γ), which is predominantly expressed in adipose tissue and contributes to decreased hepatic fat content and enhanced glycemic control and insulin sensitivity [97]. Furthermore, TZDs elevate plasma adiponectin levels and activate adenosine monophosphate [98]. Studies have elucidated the impact of TZDs on liver enzymes and histological profiles. Research in rat models has shown that pioglitazone and rosiglitazone inhibit the activation of hepatic stellate cells in vitro and ameliorate hepatic steatosis and fibrosis in vivo [99]. The primary adverse effect of TZDs is weight gain, but they can be effectively combined with metformin [100, 101].

Liraglutide, a synthetic long-acting GLP-1 receptor agonist, is approved for the treatment of diabetes mellitus and obesity. Clinical studies have indicated that liraglutide can reduce metabolic dysfunction, insulin resistance and lipotoxicity in patients with NASH without improving fibrosis [102, 103]. However, a recent study demonstrated that combining bempedoic acid, an inhibitor of liver ATP citrate lyase (ACLY), with liraglutide, reduces liver steatosis, hepatocellular ballooning and hepatic fibrosis in a mouse model of NASH [104].

### Statins

Statins have anti-inflammatory, antioxidant, and anti-fibrotic effects and reduce cholesterol biosynthesis by inhibiting HMG-CoA reductase [105]. Atorvastatin, lovastatin, simvastatin and fluvastatin are lipophilic drugs. Conversely, pravastatin and pitavastatin are hydrophilic, resulting in minimal metabolism in the liver. Studies indicate that hydrophobic statins, such as lovastatin and simvastatin, accumulate more in the liver than hydrophilic statins. However, the specific clinical implications of these differences remain unclear [105]. A comprehensive review of the safety and efficacy of statins in NAFLD patients revealed that these drugs effectively reduce liver enzyme levels and positively impact liver histology [106]. Moreover, high cumulative doses of certain statins, including rosuvastatin, pravastatin, atorvastatin, simvastatin, and fluvastatin, have been shown to reduce the risk of NAFLD-related decompensated liver cirrhosis in T2DM patients [107]. The combination of rosuvastatin and ezetimibe was found to more effectively reduce hepatic lipid accumulation than monotherapy in NAFLD patients [108]. Another primary benefit of statins is their association with decreased mortality from cardiovascular disease (CVD). Post hoc analysis of three extensive randomized clinical trials suggested that certain statins, particularly atorvastatin, not only ameliorate NAFLD/NASH but also reduce CVD events twice as in those with normal liver function [109]. Nonetheless, statins have been underprescribed in patients with NAFLD, primarily due to apprehensions about hepatotoxicity [110].

### Vitamin E

Vitamins possessing antioxidant properties are widely recognized for their health benefits. This effectiveness is attributed to their ability to reduce reactive oxygen species levels in the body via diverse mechanisms, thereby preventing oxidative cellular damage, which can culminate in cellular senescence and apoptosis [111]. Elevated transport of fatty acids to the liver during metabolic synthesis escalates oxidative stress through fatty acid oxidation and oxidative phosphorylation, creating a milieu rich in reactive oxygen species that potentially results in hepatocellular damage and progressive liver injury [112]. Vitamin E enhances the activity of the antioxidant enzymes catalase and glutathione peroxidase [113]. Furthermore, genetic factors, including variations in haptoglobin and fatty acid desaturase 1/2 (FADS1/FADS2), may influence the therapeutic efficacy of Vitamin E in NAFLD [114]. Clinical studies have demonstrated Vitamin E ameliorates liver fibrosis in patients with NASH [115]. Additionally, a combination treatment of vitamin E and hydroxytyrosol has been shown to inhibit hepatic stellate cell proliferation by disrupting the nuclear translocation/activation of SMAD2/3 transcription factors, thereby improving NAFLD-related fibrosis in children [116].

### Ursodeoxycholic acid

Ursodeoxycholic acid (UDCA) is well known for dissolving cholesterol stones, which can stabilize cell membranes, safeguard mitochondria, mitigate bile salt toxicity, and be employed in the treatment of primary biliary cholangitis and cholestatic liver disease. UDCA has demonstrated improvements in hepatic steatosis and inflammation in animal models [117, 118]. This improvement was partially attributed to the inhibition of the miR-34a/SIRT1/p53 pathway [119]. Additionally, UDCA has been shown to partially restore gut microbiota, repair gut barrier integrity, and reduce hepatic inflammation in NASH [120]. Moreover, UDCA significantly normalizes liver enzymes within the initial 3 months of treatment, enhances lipid profiles, and alleviates hepatic steatosis independently of weight loss. It also positively influences carotid intima-media thickness (CIMT) and reduces 10-year atherosclerotic cardiovascular disease risk in women following 6 months of treatment [121].

### Betaine

Betaine, a compound synthesized in the liver via oxidation of dietary choline, is a naturally occurring dietary component [122]. Betaine deficiency commonly arises from malnutrition, notably in chronic alcoholism, and is implicated in both alcoholic steatohepatitis (ASH) and NASH [123]. As a key component of the methyl donor pathway, betaine is present in carbohydrate-rich foods. However, diets deficient in choline or betaine lead to the hypomethylation of homocysteine and phosphatidylethanolamine, which in turn causes hepatic steatosis and inflammation. Evidence from a case‒control study revealed significantly lower betaine levels in patients with NASH than those with NAFLD [124]. Additionally, betaine mitigates hepatic lipid accumulation, gluconeogenesis, and inflammation by restoring LXRα and PPARα expression and reducing ER stress in fructose-fed rats [125]. Recent research indicates that betaine counteracts HFD-induced disruptions in hepatic lipid and iron metabolism by decreasing CpG methylations on the promoters of lipogenic and iron-metabolic genes [126]. Furthermore, maternal betaine supplementation was found to ameliorate MHFD-induced NAFLD, potentially by modulating gut microbiota and SCFAs in offspring mice [127].

## Non-pharmacological Treatment of NAFLD in Western Medicine

At present, there is no approved pharmacological treatment for NAFLD. Consequently, non-pharmacological interventions, including dietary modifications, exercise and bariatric surgical procedures, have emerged as crucial components in the treatment of NAFLD.

### Mediterranean diet

The Mediterranean diet (MD) is a traditional dietary pattern prevalent in European countries, characterized by high consumption of whole grains, fruits, legumes, vegetables, and seafood, moderate dairy intake, and limited consumption of meat and poultry [128]. A 6-week cross-over study revealed that adherence to the MD significantly ameliorates hepatic steatosis and diminishes insulin resistance, correlating with weight loss [128]. Moreover, adherence to the MD not only positively impacts clinical parameters such as weight, waist circumference, hepatic fat accumulation, insulin resistance, alanine transaminase levels, gamma-glutamyl transferase, total cholesterol and triglycerides, but also beneficially influences inflammatory biomarkers, including adhesion molecules and cytokines [129]. Furthermore, the new suggested strategy of the use of a green-Mediterranean diet enriched with green plant-based proteins/polyphenols such as mankai, green tea, and walnuts and reduced in red/processed meat, has been shown to decrease NAFLD incidence by half [130].

### Fitness exercise

Aerobic exercise has been demonstrated to facilitate fat breakdown in various tissues, enhance oxidative enzyme activities, activate γ receptors, and modify adipose factor levels, thereby contributing to the improvement of NAFLD [131]. A study examining the impact of different aerobic exercise intensities on 220 Chinese NAFLD patients over 6 and 12 months revealed that the experimental group exhibited enhanced liver adaptability to exercise and a decrease in hepatic fat content [132]. Additionally, the combination of intermittent fasting and exercise has proven more effective at reducing hepatic steatosis in NAFLD patients than either fasting or exercise alone [133]. Consequently, it is advisable for NAFLD patients to engage in moderate exercise when they have good nutritional status.

### Surgical

Metabolic surgery currently represents a reliable method for treating NAFLD, and procedures such as gastric banding are utilized to diminish gastric capacity and limit food intake [134]. There is substantial evidence suggesting that metabolic surgery leads to optimal results in terms of weight reduction, improvement in dyslipidemia, decreased mortality from obesity-related diseases, and enhanced glucose and lipid metabolism, as well as hepatic fibrosis mitigation. Notably, it also significantly reduces the risk of liver cirrhosis by approximately 70% [135]. Furthermore, for obese patients with NAFLD, bariatric surgery is a viable alternative characterized by a relatively low rate of perioperative complications. In patients with severe obesity accompanied by complex conditions, this approach should be considered a therapeutic option [136].

### Pharmacological treatment of NAFLD in TCM

The TCM treatment principles of NAFLD advocate phased approaches: initial treatment focuses on soothing the liver and qi, as well as fortifying the spleen and stomach, while intermediate and advanced stages emphasize enhancing the spleen and qi, resolving blood stasis, dissipating nodules, clearing heat, and detoxifying. TCM formulas are distinguished by their individualized treatment strategies, which exhibit multi-component and multi-pathway pharmacological effects in the treatment of NAFLD [137]. Given the varied syndrome types in NAFLD patients, specific prescription treatments are essential, which should be tailored to the primary symptoms while also considering secondary manifestations. Here, we introduce the classic formulas and summarize the progress of research on formulas for the treatment of different types of NAFLD (Table 1).

**Table 1:** The traditional Chinese Medicine Formulas for Treatment of NAFLD

Treatment principle	Chinese medicinal formula	Model	Mechanisms	Literature
Removing dampness and turbidity	Fu Fang Zhen Zhu Formula	STZ injection combined with HFD- induced T2DM with NASH in mice	Activating the AMPK signaling pathway	[139]
	Fu Fang Zhen Zhu Formula	HFD-induced NASH in mice	Inhibiting gut inflammation, improving intestinal barrier function, and modulating intestinal microbiota composition	[140]
Liver-soothing and spleen-invigorating	Linggui Zhugan Decoction	HFD-induced NAFLD in rats	Reducing the m6A methylation levels of suppressor of SOCS2	[142]
	Linggui Zhugan Decoction	Patients with NAFLD	Increasing DNA N6-methyladenine modification of PPP1R3A and ATG3	[143]
	Linggui Zhugan Decoction	HFD-induced NAFLD in mice	Inhibiting the STING signaling pathway	[144]
	Danshao Shugan Granules	Patients with NAFLD and HFD-induced NASH in rats	Increasing activity of superoxide dismutase and reducing NF‑κB activity	[145]
	Chaihu Shugan Powder	HFD-induced NAFLD in rats	Regulating miRNAs related to fatty acids metabolic process, such as miR-34a-5p, miR-146a-5p, miR-20b-5p and miR-142-3p	[146]
	Qinlian Hongqu Decoction	HFD-induced NAFLD in rats	Inhibiting the IRE1-α/IKKB-β/NF-κB signaling pathway	[147]
Clearing heat and removing dampness	Yinchen Wuling Powder	HFD-induced NAFLD in rats	Increasing the abundance of butyrate-producing bacteria to reduce ammonia production and promote ammonia degradation	[148]
	Gegen Qinlian Decoction	HFD-induced NASH in rats	Inhibiting the TLR4 signaling pathway	[149]
	Huangqin Decoction	HFD-induced NAFLD in rats	Blocking the TLR4/NF-κB/NLRP3 pathway	[150]
	Zexie-Baizhu Decoction	HFD-induced NAFLD in mice	Impeding lipogenesis including the SIRT1 and AMPK and promoting fatty acid oxidation	[151]
	Danshen Zexie Decoction	HFD-induced NAFLD in rats	Suppressing the ROS/NLRP3/IL-1β signaling pathway by activation of NRF2	[152]
	Qige Decoction	HFD-induced NAFLD in rats	Reducing hepatic insulin resistance and triglyceride biosynthesis via the PPP1R3C/SIK1/CRTC2/SREBP1 signal axis	[153]
	Si Miao Formula	HFHS-induced NAFLD in mice	Modulating the gut microbiota composition and increasing the abundance of Akkermansia muciniphila	[154]
Removing blood stasis and dispersing knots	Gexia Zhuyu Decoction	HFD-induced NAFLD in mice	Increasing miRNA-24 expression to decrease TRPM4	[155]
	Er Chen Decoction	HFD-induced NAFLD in rats	Regulating the gut microbiota and serum metabolism to inhibit oxidative stress and inflammation	[156]
	Qushi Huayu Decoction	HFD-induced NAFLD in rats	Downregulating intestinal MAPK pathway	[157]
	Qushi Huayu Decoction	HFD-induced NAFLD in rats	Regulating the gut microbiota to intervene in serum lipids	[158]
	Tianhuang Formula	HFD-induced NAFLD in mice	Reducing oxidative stress by increasing the abundance of Lactobacillus and its metabolite 5-MIAA to activate the NRF2	[159]
	Sanhuang Tang	HFD-induced NAFLD in mice	Activating INSR/IRS1/AKT/FoxO1 pathway	[160]
Tonifying spleen and kidney	Yiqi BuShen Tiaozhi Formula	HFD-induced NASH in mice	Regulating the expression of miRNAs, such as Mmu-let-7a-5p, mmu-let-7b-5p, mmu-let-7g-3p and mmu-miR-106b-3	[161]
	Yinchen Linggui Zhugan Decoction	HFD-induced NAFLD in rats	Inhibiting TNF signaling pathway	[162]
	Sini San	HFD-induced MAFLD in mice	Reducing YAP1 expression and lipid droplet accumulation	[163]
	Spleen-strengthening and liver-draining herbal Formula	NAFLD with patients	Regulating the disturbance of intestinal flora, especially Coprococcus, Lachnospiraceae_NK4A136 group, and Ruminococcus genus	[164]

### Removing dampness and turbidity

The primary formula for treating spleen-deficiency and phlegm-turbid stagnation is Modified Weiling Decoction from *Dan Xi Xin Fa.* It primarily comprises ingredients such as Atractylodes macrocephala, Tangerine peel, Magnolia officinalis, Glycyrrhiza glabra, Polygonum multiflorum, Poria cocos, Bupleurum falcatum and Cinnamon. Ingredient modifications of these formulas based on clinically specific symptoms: Gynostemma and Hawthorn are added for those who were obese and had apparent surrounding dampness. Modified Weiling Decoction has been found to effectively reduce the levels of TG, TC, ALT and AST in the serum of patients with NAFLD [138].

The Fu Fang Zhen Zhu Formula (FTZ) improves hepatic steatosis and fibrosis in NAFLD with type 2 diabetes by activating the AMPK signaling pathway [139]. In addition, FTZ ameliorates NASH by inhibiting gut inflammation, improving intestinal barrier function, and modulating the intestinal microbiota composition [140].

### Liver-soothing and spleen-invigorating

The primary formula for treating stagnation of liver-qi is Xiao Yao Powder from the *Tai Ping Hui Min He Ji Ju Fang*. It primarily comprises ingredients such as Angelica sinensis, Paeonia alba, Bupleurum, Poria, Atractylodes macrocephala, Glycyrrhiza, Gentian and Peppermint. Ingredient modifications of these formulas were performed based on clinically specific symptoms: Citrus Medica and ginger peel are added for obvious abdominal distension; Mongolian milkvetch and Codonopsis pilosula are added for short-term fatigue. A meta-analysis based on 12 studies with 1012 participants suggested the potential therapeutic effect of Xiao Yao Powder for NAFLD [141].

Linggui Zhugan Decoction alleviates hepatic steatosis through cytokine signaling 2 modification by N6-methyladenosine in mice and improves insulin resistance in overweight/obese patients with NAFLD through regulating the DNA N6-methyladenine modification of phosphatase 1 regulatory subunit 3A and autophagy related 3 [142, 143]. Moreover, Linggui Zhugan Decoction ameliorates HFD-induced hepatic lipid deposition in mice by inhibiting STING-mediated inflammation in macrophages [144]. Danshao Shugan Granules had significant effects on total cholesterol, triglyceride, aspartate transaminase and γ-glutamyl transpeptidase in NAFLD patients [145]. Chaihu Shugan Powder inhibits the expression of 15 miRNAs, such as miR-34a-5p, miR-146a-5p, miR-20b-5p and miR-142-3p, and fatty acid biosynthesis related enzymes, thus reducing fatty acid biosynthesis in NAFLD [146]. Qinlian Hongqu Decoction inhibits the IRE1-α/IKKB-β/NF-κB signaling pathway in the liver, thereby attenuating inflammation and insulin resistance in NAFLD rats [147].

### Clearing heat and removing dampness

The primary formula for treating accumulated damp-heat is Yinchen Wuling Powder from *Jin Kui Yao Lue*. It primarily comprises ingredients such as Capillaris Herba, Alismatis Rhizoma, Atractylodes Macrocephalae Rhizoma stir-fried with wheat bran, Poria and Cinnamomi Ramulus. Ingredient modifications of these formulas were performed based on clinically specific symptoms: Ginger pinellia and Bamboo shavings are added for Jaundice; Polygonum cuspidatum is added for those trapped by damp-evil. Yinchen Wuling Powder shows hepatoprotective effects by regulating the imbalance of the gut microbiota and increasing the abundance of butyrate-producing bacteria to regulate pro-inflammatory cytokines and immune function [148].

Gegen Qinlian Decoction inhibits the TLR4 signaling pathway to reduce ALT, AST, liver tissue IL-6, and TNF-α levels to reduce liver injury and improve NASH [149]. Huangqin Decoction ameliorates hepatic inflammation in NAFLD rats by blocking the TLR4/NF-κB/NLRP3 pathway [150]. Zexie Baizhu Decoction impedes lipogenesis and promotes fatty acid oxidation to alleviate lipid disorders and protect the liver in NAFLD [151]. Danshen Zexie Decoction suppresses the ROS/NLRP3/IL-1β signaling pathway by activating Nrf2 to reduce lipid accumulation and alleviate oxidative stress, inflammation and pyroptosis in NAFLD rats [152]. Qige Decoction inhibits hepatic steatosis by reducing hepatic insulin resistance and triglyceride biosynthesis via the PPP1R3C/SIK1 signaling axis [153]. Si Miao Formula attenuates NAFLD, which is linked to the modulation of the gut microbiota composition, especially that of Akkermansia muciniphila[154].

### Removing blood stasis and dispersing knots

The primary formulas for treating stasis blocking channels are Gexia Zhuyu Decoction, sourced from *Yi Lin Correction,* and Er Chen Decoction, derived from *Tai Ping Hui Min He Ji Prescription*. These formulas primarily include ingredients such as Peach kernel, Peony bark, Radix paeoniae rubra, Solanum nigrum, Ligusticum wallichii, Angelica sinensis, Safflower, Coix lacryma, Poria and Ginger. Ingredient modifications of these formulas were performed based on clinically specific symptoms: Curcuma longa and Ginger are added for blood stasis, such as a dark face. Gexia Zhuyu Decoction has been found to modify miRNA-24 and transient receptor potential melastatin 4 (TRPM4) expression, thereby enhancing NAFLD treatment efficacy in mice [155]. Er Chen Decoction alleviates NAFLD in rats by preventing oxidative stress, diminishing inflammatory responses, and ameliorating gut microbiota dysbiosis, as well as modulating metabolites in serum, including taurine and hypotaurine, cysteine and methionine and vitamin B6 [156].

Qushi Huayu Decoction reduces liver injury in NASH by suppressing the MAPK pathway to protect the colonic tight junction and alters serum lipids by regulating the gut microbiota, including Bacteroides, Blautia, Lachnoclostridium and Turicibacter [157, 158]. Tianhuang Formula increases the abundance of Lactobacillus and the content of 5-MIAA in the intestinal contents and liver to reduce the expression of Nrf2 and oxidative stress, thereby improving NAFLD [159]. Sanhuang Tang alleviates insulin resistance by activating the INSR/IRS1/AKT/FoxO1 pathway, which contributes to NAFLD treatment [160].

### Tonifying spleen and kidney

The primary formulas for treating spleen and kidney deficiency are Si Jun Zi Decoction, sourced from *Tai Ping Hui Min He Ji Ju Fang*, and Jin Kui Shenqi Pill, derived from Jin Kui Yao Lue. These formulas primarily comprise ingredients such as Ginseng, Poria, Atractylodes, Licorice, Rehmannia, Cornu Cervi pantotrichum, Yam, and Peony peel. Ingredient modifications of these formulas are performed based on specific clinical symptoms: Boswellia root and Eucommia are added for waist and knee soreness, dizziness and weakness; Radix pseudostellariae and Cinnamon are added for cold extremities; Radix chinensis and Cuttlebone are added for frequent nocturnal urination; Fried lentils and Fried coix seeds are added for diarrhea.

Yiqi BuShen Tiaozhi Formula primarily improves NASH by modulating the expression of various miRNAs, such as mmu-let-7a-5p, mmu-let-7b-5p, mmu-let-7 g-3p, and mmu-miR-106b-3p [161]. Yinchen Linggui Zhugan Decoction effectively inhibits the expression of the TNF signaling pathway and alleviates HFD-induced liver injury and inflammatory response in rats with NAFLD [162]. Sini San reduces lipid droplet accumulation and YAP1 expression in hepatocytes, and the knockout of hepatocellular YAP1 reduces the effect of Sini San on reducing lipid deposition [163]. Spleen-strengthening and liver-draining herbal Formula can improve liver function and glucolipid metabolism in patients with NAFLD by regulating the disturbance of intestinal flora, including Coprococcus, Lachnospiraceae and Ruminococcus genus [164].

## Non-pharmacological Treatment of NAFLD in TCM

### Acupuncture

Acupuncture, a widely-used adjunctive therapy in TCM, originates from China and boasts a history exceeding 4000 years. It is employed to promote health and treat diverse diseases via techniques such as manual needling, electroacupuncture, moxibustion, and acupressure at specific anatomical sites, termed acupoints [165]. Numerous studies have demonstrated the efficacy of acupuncture in treating NAFLD, notably in impacting liver fat, lipid metabolism, and insulin resistance [166]. Recent research underscores the beneficial role of traditional acupuncture therapy in treating NAFLD, as it provides convenient treatment with minimal adverse reactions and considerable efficacy [167].

Acupuncture can inhibit the progression of NAFLD by suppressing inflammation, mitigating oxidative stress, and enhancing lipid metabolism in liver cells [166]. A study revealed that electroacupuncture at the bilateral acupoints “Spleen Shu,” “Kidney Shu,” and “Diaphragm Shu” effectively decreased the expression of TNF-α in NAFLD rats, thereby ameliorating the inflammatory state of NAFLD and reducing liver damage [168]. Furthermore, acupuncture has been shown to significantly enhance lipid metabolism and the systemic inflammatory response in HFD-induced NAFLD rats, possibly through the regulation of the intestinal microbiota composition [169]. Numerous clinical studies have indicated that acupuncture is effective at lowering total cholesterol (TC), TG, and low-density lipoprotein cholesterol (LDL-C) levels and increasing high-density lipoprotein cholesterol (HDL-C) levels, thus improving lipid metabolism in patients with dyslipidemia [170].

### Acupoint embedding

Acupoint embedding is an improved technique based on traditional acupuncture intervention. The core principle of this technique entails the placement of absorbable sutures, such as sheep gut sutures, within acupoints to extend the stimulation duration and augment efficacy, thus diminishing the necessity for frequent interventions. Additionally, acupoint embedding offers inherent practical convenience [171]. Extensive clinical research has been undertaken to establish the efficacy and safety of acupoint embedding therapy in the treatment of NAFLD [172]. Studies have shown its superior effectiveness in treating NAFLD with abnormal liver enzyme levels compared with traditional pharmacotherapy [173]. Significantly, a meta-analysis confirmed the lipid-lowering impact of acupoint embedding in NAFLD [174].

### Martial arts

Traditional Chinese martial arts, such as Tai Chi, have demonstrated therapeutic efficacy as a form of physical exercise in the treatment of metabolic diseases [175]. In a recent study, Tai Chi was observed to significantly decrease waist circumference and elevate high-density lipoprotein (HDL) cholesterol levels in obese adults [176]. Furthermore, Tai Chi has been associated with reductions in waist circumference, body mass index (BMI), blood glucose levels, and insulin resistance in T2MD patients [177]. These studies suggest the potential of Tai Chi in the treatment of NAFLD. Interestingly, Tai Chi has been also found to be more effective than conventional exercise in reducing anxiety and depression, as well as in enhancing overall mental health [178]. This distinction highlights the unique aspects of Tai Chi, which combines physical movement with mindfulness and deep breathing techniques. The practice's holistic approach not only addresses physical well-being but also significantly impacts psychological health, offering a more comprehensive benefit compared to traditional forms of exercise that primarily focus on physical fitness.

## Integration of TCM and Western medicine for the treatment of NAFLD

The current research on the integration of TCM and Western medicine for the treatment of NAFLD is still in early stages. However, there is already some evidence indicating that this integrative approach may offer superior treatment outcomes for NAFLD. A study indicates that combining Xiao Yao San with simvastatin enhances treatment efficacy compared to simvastatin alone, significantly improving liver function and lipid profiles in NAFLD patients [179]. Similarly, a combination of Compound Danshen Tablets and metformin for treating diabetes with NAFLD shows greater improvement in liver function, blood sugar, and lipid levels than using metformin alone [180]. Additionally, electroacupuncture combined with lifestyle modifications has been found to be more effective than lifestyle modifications alone in treating obesity-related NAFLD, improving liver fat status, glycemic control, and insulin resistance [181]. While the integration of TCM and Western medicine shows promise in treating NAFLD, more research is needed to fully understand its efficacy. Future research should focus on large-scale clinical trials to validate these findings and guide clinical practice in the holistic management of NAFLD.

## Conclusions

In the foreseeable future, Chinese medicine is poised to become a significant source of potential therapeutics for the treatment of NAFLD, although realizing this vision entails overcoming considerable challenges. The first challenge involves translating traditional Chinese medicine terminology into a universally recognized scientific language and establishing a standard, repeatable method for identifying the contents of various Chinese herbs, facilitating improved recognition and communication between Chinese and Western medicine. The second challenge entails conducting rigorous clinical research on TCM for the treatment of NAFLD and elucidating the benefits of evidence-based approaches in this context. The third challenge involves conducting comprehensive research on the mechanisms of action of Chinese formula medicines in NAFLD and verifying whether their observed effects can be optimized through a scientifically robust, practical, and accredited validation platform. However, finding the precise target for improving NAFLD through molecular research is challenging due to the diverse compositions of Chinese herbal medicines. The current strategy is dissecting the molecular mechanisms of the prescription by analyzing individual herbs or active ingredients. However, this approach deviates from the principles of syndrome differentiation in TCM.

In the management of NAFLD, both TCM and Western medicine offer distinct approaches with respective advantages and limitations. Western medicine, grounded in clinical trials and scientific research, provides evidence-based treatments such as antidiabetic drugs (e.g., Metformin) and antioxidants (e.g., Vitamin E), which can improve liver function and reduce hepatic fat. However, these treatments may come with side effects like gastrointestinal symptoms and weight gain, and are often limited in reversing the progression of advanced fibrosis stage. Conversely, TCM focuses on holistic balance and regulation, utilizing herbal medicine and acupuncture to treat NAFLD. While generally associated with fewer side effects and suitable for long-term use, TCM's efficacy and mechanisms require further validation through rigorous clinical studies, and treatments may vary significantly among individuals. In summary, Western medicine is offering rapid symptom amelioration and TCM is enhancing overall bodily function, possibly reducing the side effects of conventional drugs and potentially improving overall therapeutic outcomes. Investigations into the integration of TCM and Western medicine for the treatment of NAFLD are nascent and require further investigation and dedication. This emerging field could potentially shape the future direction of NAFLD prevention and management. However, the effectiveness and optimization of such integrated therapeutic strategies necessitate rigorous scientific inquiry and clinical trials.

## Data Availability

Not applicable.

## Abbreviations

AMPK: AMP-activated protein kinase
AKT: Protein Kinase B
ATG3: Autophagy-related proteins 3
BA: Bile acid
CRTC2: CREB-regulated transcription coactivator2
FoxO1: Forkhead box protein O1
GCKR: Glucokinase regulatory protein
HFD: High-fat diet
INSR: Insulin receptor
IRE1-α: Inositol requiring enzyme 1-α
IRS1: Insulin receptor substrate 1
IKKB: IkBa kinase B
L-1β: Interleukin-1β
LPS: Lipopolysaccharides
MAPK: Mitogen-activated protein kinases
MBOAT7: Membrane-bound O-acyltransferase domain-containing 7
NAFL: Non-alcoholic fatty liver
NASH: Non-alcoholic steatohepatitis
NF‑κB: Nuclear factor-κB
NLRP3: Nod-like receptor family pyrin domain-containing 3
NRF2: NF-E2-related factor 2
PI3K: Phosphoinositide 3-kinase
PNPLA3: Patatin-like phospholipase domain-containing 3
PPP1R3A: Protein phosphatase 1 regulatory subunit 3A
PPP1R3C: Protein phosphatase 1 regulatory subunit 3C
ROS: Oxygen species
SCFA: Short-chain fatty acid
SIRT1: Sirtuin 1
SIK1: Salt-inducible kinase 1
SOCS2: Cytokine signaling 2
SREBP1: Sterol regulatory element binding protein 1
STING: Stimulator of interferon genes
STZ: Streptozotocin
T2DM: Type 2 diabetes mellitus
TM6SF2: Transmembrane 6 superfamily member 2
TNF: Tumor necrosis factor
TLR4: Toll-like receptor 4
TRPM4: Transient receptor potential melastatin 4
YAP1: Yes-associated protein 1
